# A Critical Exploration of Child-Parent Attachment as a Contextual Construct

**DOI:** 10.3390/bs8120112

**Published:** 2018-12-11

**Authors:** Ya-Hsin Lai, Sam Carr

**Affiliations:** Department of Education, University of Bath, Bath BA2 7AY, UK; S.Carr@bath.ac.uk

**Keywords:** attachment, parent-child relationship, parenting, contextual, context-specific, sport, academic, hierarchical model

## Abstract

Bowlby’s attachment theory has been employed as a broad and integrative framework to explore human wellness across a range of disciplines. Attachment theory has even been labelled one of the last surviving “grand theories” not to have been completely dismissed, replaced, or extensively reworked. However, despite the ubiquitous nature of some of the theory’s fundamental tenets, there are always possibilities for new conceptual development, extension, and revision. In this paper, we critically explore the idea of “context-specific” attachment within parent-child relationships. We briefly outline critical assumptions and key areas of attachment and articulate potential rationale, conceptualization, and relevance of contextual attachment.

## 1. Basic Tenets of Attachment Theory 

Bowlby [1] drew upon the notion of behavioral systems (based upon the idea of biologically evolved neural programs) to describe the processes by which human beings organize behavior in response to inevitable environmental changes and demands to maximize chances of survival and reproduction. According to Bowlby’s [1] propositions, the biological function of the attachment system ensures that infants seek out a stronger, wiser, and protective attachment figure for proximity maintenance and protection, support, and care, especially during dangerous or difficult situations (see also Mikulincer and Shaver [2]). Normally, when individuals encounter environmental threats or stressors, the attachment system is activated to secure care or protection from selected caregivers. When these systems are deactivated or when dangers/threats are not present, the attachment system is quietened, and psychological energy can be devoted to exploration or other activities [1]. Specifically, obtaining a sense of security is the goal of such attachment behavior (especially when encountering actual or symbolic threats and/or where a reliable caregiver is not available or responsive) and the attainment of “felt-security” deactivates further attachment-related efforts (see Sroufe [3]). The process of experiencing a sense of security can, over time, help to develop a prototypical “secure base script” around key issues such as the possibility of coping with threat, obtaining care and support, and managing negative emotion in future interpersonal relationships [4]. 

When a selected caregiver fails to meet needs for comfort and care during times of distress, then the primary attachment strategy is unable to accomplish the goal of felt-security. In such cases, the attachment system can be adjusted and certain secondary attachment strategies (e.g., hyper-activation and deactivation) are likely to be activated in accordance with situational demands [5,6]. For instance, a person may adopt hyper-activation strategies, such as intensifying proximity-seeking efforts, to secure love, care, and attention from caregivers and to deal with frustrated attachment needs. Deactivation strategies (labelled “compulsory self-reliance” by Bowlby), on the other hand, tend to involve the suppression of attachment needs. Normally, an individual learns to use deactivation strategies to deal with threat and distress and to avoid the disappointment, frustration, and pain that comes from lack of caregiver availability [1].

Ainsworth [7] initially conceptualized a child’s interactions with the primary caregiver into three major attachment styles—secure, insecure-anxious, and insecure-avoidant. Such prototype-like attachment styles reflect the most chronically accessible working model. Children with a secure attachment relationship with the primary caregiver usually hold advantageous working models of successful proximity seeking and attainment of security because of predominantly attentive, empathic, and supportive responses to emotional needs, especially during vulnerable moments. Children who receive such secure responses from parents may consider themselves worthy of being loved by others and feel confident and able to seek support and emotional relief from parents when they feel upset, threatened, or stressed [8]. In contrast, a child classified as insecure-anxious tends to access working models of attachment characterized by hyper-activation to acquire the goal of felt-security. Typically, anxious children’s maladaptive attachment behaviors are the reflection of inconsistent and lacking responses to seeking emotional support [8]. Children with insecure-avoidant attachment models tend to deactivate security-seeking behavior and have typically experienced significant neglect, rejection, and unresponsiveness in relation to proximity-seeking attempts [8]. 

The research on attachment has diverged into two distinct research “traditions” [9]. These lines of research are both derived from the assumptions at the heart of Bowlby’s theory [10], yet have evolved according to underlying assumptions and measurement techniques of contrasting subcultures [11]. Many of the distinctions between these two lines of enquiry are reflected in how researchers have approached the measurement of attachment constructs. On the one hand are researchers who “… tend to think psychodynamically, be interested in clinical problems, prefer interview measures and behavioral observations over questionnaires, study relatively small groups of subjects …” [11] (p. 27). On the other hand are personality and social psychologists “… who tend to think in terms of personality traits and social interactions, be interested in normal subject populations, prefer simple questionnaire measures, study relatively large samples …” [11] (p. 27). Not surprisingly, these different lines of research give rise to significant distinctions in terms of how attachment research is conceptually underpinned, how attachment is measured and how results are interpreted. We conceptualize contextual attachment characteristics in a social psychological sense (self-report paradigm) as the basis for this article.

## 2. Continuity, Stability, and Fluctuation of Attachment Styles

The stability and change of internal working models of attachment have been broadly explored and discussed in the literature (e.g., [12,13,14,15,16]). Understanding and exploring fluctuation in attachment styles across the lifespan is conceptually challenging and highly complex. Initially, Bowlby [17] argued that attachment representations can be spontaneously operated by both processes of “assimilation” and “accommodation”, where individuals not only integrate new experiences into existing mental representations but also revise previous working models to accommodate current attachment associative experiences.

For example, some attachment theorists (e.g., [13,18]) have proposed a “prototype perspective”, suggesting that there are two separate working models (“prototype-like” and “current” working models) that concurrently function to shape a person’s “phase-specific” attachment characteristics. From this perspective, a person’s “current working models” can be revised and updated throughout the lifespan when “present experiences” of attachment deviate from prototypical attachment beliefs and knowledge that have been formed in childhood (a “prototype working model” is thought to be rooted in a person’s infancy). In other words, while a person’s “prototypical working model” plays a fundamental and prevailing role in retaining early attachment trends, such models can still incorporate incompatible attachment experiences from later developmental phases and present experiences, resulting in structural/qualitative changes in phase-specific attachment schemata. For instance, when securely attached adolescents (who may have developed secure working models during infancy and childhood) frequently experience being rejected or neglected by attachment figures, their existing security may be compounded by these continually conflicting experiences and memories [19] (p. 112).

Such a view tends to be favored in contemporary research and is sensible to explain both the fluctuation of attachment throughout the lifespan and the inconsistent research in relation to continuity of attachment characteristics [13,19]. According to Fraley’s [13] meta-analysis of attachment stability from infancy to adulthood, there is a moderate level of association (0.39) between attachment orientations across different developmental stages (especially up to 19 years old). This result seems to be in line with other research (e.g., [20,21,22]) that has found a moderate correlation between early attachment security with parents and attachment in later adult relationships, suggesting that prototypical attachment styles do not completely set the tone for attachment through the lifespan. 

### 2.1. Multiple Working Models in Relational Networks

With age, the expansion and extension of social and relational life can be (but is not always) conducive to the formation of a wider variety of attachment bonds with multiple figures (such as grandparents, older siblings, neighbors, relatives, close friends, teachers/coaches, coworkers, romantic partners, and spouses) as subsidiaries for closeness and sources of security [12,23,24,25,26]. These “attachment figures” tend to be relationship referents who serve some or all of the functions of proximity maintenance, safe haven and secure base provision. However, in adolescence, parents—compared to other relational figures—remain important, chronic, and influential figures in the attachment hierarchy [27,28,29,30].

Previous studies have suggested that the role of “principal” attachment figure can change according to developmental level. For example, parents are the most likely primary attachment figures until late childhood, whereas close friends and romantic partners can become the preferred and prevailing attachment figures for many adolescents and adults [28,31,32,33]. This does not necessarily mean that parents no longer serve as attachment figures per se, simply that individuals’ attachment hierarchies expand and develop, often meaning that different roles and attachment functions (i.e., proximity, safe-haven, and secure-base functions) are served by different attachment figures [27,28,34,35,36]. Furthermore, research (e.g., [35,37,38,39]) has suggested that individuals’ attachment-related needs may vary dramatically between relationships or relational domains (e.g., familial, friendship, romantic). 

La Guardia et al. [40] explored within-person variation in attachment security across a range of relationship referents (e.g., mother, father, romantic partner, best friend). Through a self-determination theory lens, they contended that the satisfaction of basic psychological needs for relatedness, autonomy, and competence in a given relationship would determine the extent to which that relationship would reflect a secure attachment bond. If such patterns of need satisfaction varied between relationships (and within-person), then it was hypothesized that there would be variability in felt attachment security between relationships. Results indicated that variability of need satisfaction in different relationships at the within-person level accounted for approximately twice as much variance in attachment variables than between-person variability. Hence, people seem to have different attachment security and models of attachment for the different attachment figures in their networks, and the greater the satisfaction of specific psychological needs in a given relationship then the greater the felt attachment security within that relationship. Such research strongly suggests that attachment security varies across the network of close relationships that individuals develop.

Research into the fluctuation and stability of people’s attachment security seems to be in line with Bowlby’s initial proposition: the formation of attachment characteristics seems to involve interactions with multiple attachment figures, which are assimilated into and help to amend experiences (or mental representations) with parents during early developmental stages and which may have some enduring influence across the lifespan but still be open to change. Attachment experiences with new relational partners are likely to serve as crucial antecedents for change in relation to a person’s attachment security and may help to form a widening pool of mental representations within specific close relationships.

### 2.2. How Does Attachment to Multiple Figures Work? 

The issue of how relationship-specific, domain-specific, and global attachment representations work together in a hierarchy of working models within a relational network has been explored by many researchers (e.g., [28,35,37,39,41,42]). Collins and Read [43] argued that it is likely that people can hold distinct attachment representations for specific relationship referents in their lives (e.g., mother, father, romantic partner) but that these attachment representations are likely to be hierarchical in terms of their fundamental importance and impact on global well-being and personality development. That is, perhaps certain relationships carry more weight in relation to the influence they have on people’s general attachment-related cognition, affect, and behavior. This may be a function of factors such as the literal amount of time spent with a given other (e.g., children are likely to spend a lot of time with parents, or sports coaches if they are athletes) or the degree of psychological investment in a relationship. It may also be that at different phases of the lifespan, different relationship-specific representations of attachment are more likely to be in flux or open to influence. Collins and Read [43] argued that by early adulthood most people’s attachment representations of their parents have been entrenched and reflect well organized sets of expectations and beliefs that are firmly established. In contrast, perhaps relationships with romantic partners are, at this stage, less entrenched and therefore likely to be malleable and more “state-like” in the way that they are experienced.

Pierce and Lydon [39] found that individuals with insecure (but not secure) attachment at the global level exhibited variability (in the quality and intimacy of social interactions) across different relationship-specific working models, suggesting that globally insecure individuals were still able to “find” security in some specific relationships, despite their global insecurity. Imamoğlu and Imamoğlu [42] also revealed that individuals reporting higher attachment security at the global level did not necessarily experience the same perceptions of attachment security across specific close relationships, again suggesting that individuals are able to form relationship-specific attachment representations that deviate from their crystallized global models. 

Overall et al. [37] have suggested that people’s attachment representations in relationship “domains” (e.g., family, friends, romantic relationships) seem to be abstract reflections of the interactions between their “global” and “relationship-specific” working models. They conceptualized that relationship-specific life events (e.g., divorces, break-ups, or affairs) would be likely to have a much greater and direct impact on the attachment representations pertaining to the specific “domain” in which they occurred and a lesser effect on other relational domains (i.e., security in romantic relationships would be affected by divorce or affairs but friendships would not). Building on previous findings (e.g., [28,35,39]), Overall et al.’s [37] data indicated a “multilevel” network of attachment representations, in which global, overarching attachment schemata (at the uppermost level) serve to orchestrate and shape generally low cognitively-accessible or ambiguous information across relational domains and integrate the most consistent experiences. However, at the midlevel tier, nested underneath global representations, are “domain-specific” models (like familial, friendship, or romantic relationships), providing more accurate differentiation of attachment-related beliefs and expectations across domains. Nested underneath these “domain-specific” models, it is proposed that relationship-specific attachment representations with multiple, specific figures (e.g., one’s mother, father, brother, close friend, and specific romantic partners) may exist. The data indicate, as one might expect, that attachment to multiple figures is likely to be complex and intricate in terms of how it is experienced and orchestrated.

## 3. The Idea of Hierarchical Attachment Representations “within” Specific Relationships: Global, Contextual, and Situational Levels

**Figure 1:**
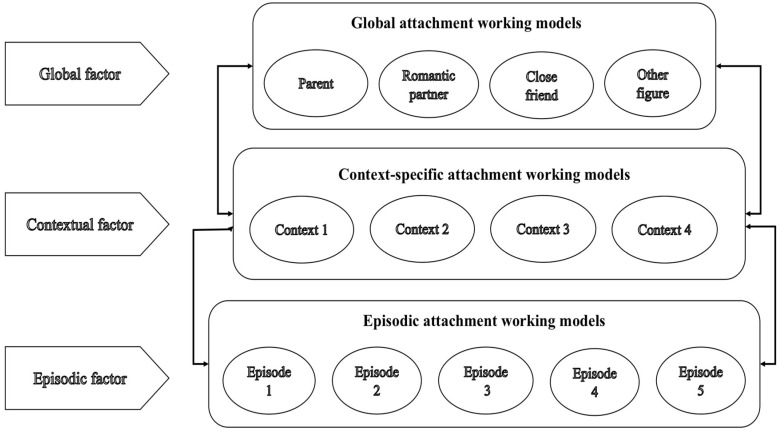
A schematic depiction of the hierarchical structure of attachment representations within specific relationships.

Existing literature has devoted significant attention to exploring the relationship between multiple attachment representations across global, domain-specific, and relationship-specific hierarchies. However, less conceptual attention has been devoted to variation in attachment patterns “within” a single attachment relationship. Gillath, Karantzas, and Fraley [43] proposed a revised hierarchical structure to add an additional level of specificity that would be nested underneath the “relationship-specific” attachment models described above. Specifically, they claimed that a person’s attachment representations might vary from moment to moment, although individual interpersonal “moments” or interactions that happen within a specific relationship and somehow share common associations would rise to relationship-specific models. Hence, we believe that even within specific relationships, a multilevel structure might be proposed that includes a generalized model of the given relationship, a model of the given relationship as it is experienced across different contexts, and a state-like fluctuation that functions episodically (see Figure 1). 

Based upon Gillath et al.’s [43] research, transient attachment-relevant interactions or “moments” within a specific relationship, and at a given time, can form “episodic” representations at the lowest level of a relationship-specific hierarchy. Episodic factors may temporarily shape attachment representations (e.g., beliefs, goals, behavioral strategies) with a given relationship partner, thereby giving rise to episodic attachment representations. For example, being cheated on by a partner may cause a loss of trust for that partner, thereby momentarily enhancing attachment insecurity within the given relationship. At the next level, we suggest that it may be important to consider “contextual” representations within a given relationship as well, which might be referred to as a series of repeated momentary episodes that cluster around a given context and seem to relate to meaningful contextual variability within a given relationship. For instance, within a given parent-child relationship there may be particular parenting behaviors attached to a given context (e.g., sport or school) that trigger or shape individuals’ attachment representations with the parent in that specific domain but not in other contexts where interactions with the same parent occur. Furthermore, individuals’ orientations at a specific level within a given relationship may be shaped by the lower/higher order level (i.e., a top-down and/or bottom-up effect) as postulated in previous hierarchical models (see [37,41,43,44,45,46]).

## 4. Contextual “Child-Parent” Attachment Representations: Conceptualization and Significance

We believe that within a given relationship, individuals could develop “context-specific” attachment schemata in relation to a specific relationship partner. Context-specific schemata could then act as mediators to connect the global and episodic levels of specificity by means of top-down and bottom-up operations. Research has indicated that throughout the lifespan individuals are capable of developing various context-specific (e.g., school-specific, sport-specific, community-specific) attachment bonds with a variety of relationship partners, including parents, close friends, teammates, teachers, coaches, and romantic partners [12,23,47,48]. This is often because these significant others are more accessible, attainable, and able to satisfy specific attachment functions (e.g., proximity, safe haven, and secure base) in a given context and at a given developmental stage [27,34,36].

Context-specific representations of attachment might be referred to as schemata in which one’s attachment representations with (for example) parents specifically vary by context (e.g., sport or school) and are stored and experienced as such in a psychological and emotional sense. As mentioned earlier, these contextual schemata could also involve interplay between contextual factors, global structures (i.e., more prototypical schemata for parents), and episodic (i.e., episodic interactions from moment to moment) representations. In other words, through extracting attachment-relevant information related to a given context, a person’s context-specific representations with parents could reflect a variety of cognitively accurate and accessible knowledge relating to that context and which is distinct from other contexts.

### 4.1. Why Should Child-Parent Attachment Representations Vary Across Contexts?

What kinds of contexts might have the capacity to shape and sculpt a contextual-level child-parent attachment representation that differs from that representation in other contexts? To some extent the answer to this question depends heavily upon the individual-difference, family, and cultural factors. It has also been suggested that various significant others (e.g., parents, coaches, teachers, colleagues) and their involvement with individuals in specific contexts (e.g., school, sport, work) may vary by developmental level and gender (e.g., [49,50,51,52]). However, one might crudely sketch out plausible “contexts” or “domains” that meaningfully connect to children’s lives. For example, many Western children’s lives revolve around contexts such as school and/or extracurricular activities like sport, art, or music [32,53,54,55] and previous research has shown a great deal of interest in the mechanisms behind parental influence on well-being in specific contexts like school and sport [49,56,57,58,59]. 

For instance, in the specific contexts of school and sport, research (e.g., [60,61]) has strongly suggested that parental belief systems in relation to a child’s ability and their subject evaluation of children’s successes and failures serve as influential “contextual cues” that shape children’s beliefs, affective patterns, and behavioral responses in a given context. Environmental characteristics (e.g., highly public, competitive arenas, evaluation/reward systems, interpersonal complexity) emphasized in contexts such as school or sport are likely to induce parental focus on specific goals and expectations for their children, and this has been shown to influence psychological outcomes (e.g., enjoyment, cognitive anxiety, attention, needs satisfaction) [62,63,64]. In short, there are reasons to believe specific contexts have the capacity to fundamentally alter the quality of parent-child interactions to the extent that they may constitute dramatic shifts in the nature of the child-parent attachment relationship.

In the sporting literature, parents who create a “performance-oriented” motivational climate, in which recognition, praise, evaluation, and value are attached to children’s demonstration of ability and superiority, are more likely to resort to controlling practices in their interactions with children. Children exposed to this motivational atmosphere have been shown to experience thwarted needs for autonomy, competence, relatedness, and associated negative emotions (e.g., anxiety, stress, pressure), especially when they are not able to meet parental requirements [55]. These performance-approach oriented motivational, cognitive, and affective cues could certainly activate and help to foster sport-specific contextual child-parent attachment representations. However, these sport-specific attachment representations need not necessarily be salient with the same parent in academic or other contexts where secure attachment interactions may be found. This may be an example of how motivational climates function as unique contextual cues to trigger context-specific attachment schemata within parent-child relationships. 

Research in other performance contexts have identified that some types of parental involvement in performance contexts can invade, interrupt, and be incompatible with fundamental aspects of a caring bond. For example, Rapport and Meleen [65] examined child-parent bonds in a sample of adults who had shown early talent in the field of screen acting and had been considered “child celebrities” between the ages of 6 months to 18 years. Of interest in this study was the nature of the self-reported parent-child relationship in child celebrities whose parents had also served as their child’s manager. Data suggested that former child performers whose parents (it was almost exclusively mothers who had fulfilled this role in the investigated sample) had served as their professional manager viewed the parental figure as less caring and more controlling than did performers whose caregivers were not their managers. The researchers argued that their data hint that the inherent role of managing a child celebrity may conflict with many of the fundamental aspects of caregiving typically associated with the parent-child relationship. For example, “managing” a child performer may require parents to adopt a more emotionally distant and objective perception of the child (e.g., in the managerial role perhaps the child is viewed as a “source of income” or as “the means to an end”) that is incompatible with features of a caring and secure parental bond. Some of these conflicts related to parental roles have also been identified in parent-coach/child-athlete dyads in the context of sport (e.g., [66]). Hence, there is reason to believe that certain contexts have the capacity to encourage and foster specific representations of attachment in child-parent bonds that may or may not be carried over into other contexts. 

The concepts of parental conditional regard (PCR) and achievement by proxy distortion (ABPD) have also been considered as maladaptive parenting practices, especially in the context of sport and school [58,59,67,68,69,70]. These achievement domains seem to be potential platforms for the demonstration of PCR and ABPD as context-specific socializing practices. Specifically, “parental conditional positive regard (PCPR)” is thought to exist when parents are perceived to offer more affection, recognition, and attention than usual when the child meets their expectations and desired aims. In contrast, “parental conditional negative regard (PCNR)” is when parents are perceived to withhold or give less affection, love, and esteem than they usual do when the child does not meet their expectations. PCPR/PCNR have been identified as disruptive parenting practices linked to significant psychological costs (e.g., introjected regulation, unstable self-esteem, negative emotions, poor relationships and well-being) [59,71,72]. It may be that, as Assor, Kanat-Maymon, and Roth [72] have claimed, children introjecting the desired behaviors and goals of their parents is a way of preventing the loss of parental appreciation or increasing the attention and love they receive from parents. However, the desire or pressure to avoid feeling unworthy or to obtain self-regard may also result in a dampened sense of autonomy [73]. Given the fact that PCR has been considered as a “domain-specific” socializing strategy for bolstering contingent introjection [72,74,75], it is plausible that context-specific PCR might serve as a contextual cue that elicits predominantly insecure child-parent attachment schemata in a given context. 

“ABPD” may be another mechanism by which parents execute “context-specific” maladaptive socializing practices in children’s achievement domains (especially in sport) (e.g., [58,67]). As an example, sport can be a competitive and reward/evaluation-focused context in which the demonstration of ability is important and emphasized by significant others. The unique characteristic and atmosphere of sport is an open door to aggressive and ambitious parents, vulnerable to ABPD pressures, especially when parents place their self-worth on a child’s success and failure in sport. Objectification of a child is one of the mechanisms of parental “achievement by proxy” in Tofler et al.’s [61] proposed ABPD spectrum. That is, parents may come to regard their children as an object, rather than a person, as a means to indirectly satisfy their own needs for achievement. This controlling parental behavior may drive a child to succeed to please parents or feel valued. However, it may also lead children to feel guilt or lose self-value if they cannot meet parents’ expectations and requirements. This introjection of parental objectification, thwarting one’s psychological needs for autonomy, competence, and relatedness in sport, could serve as an influential contextual cue to activate insecurely “sport-specific” attachment representations. 

### 4.2. Why Might “Contextual” Attachment within Child-Parent Attachment Relationships Matter?

Recent research exploring child-parent attachment and children’s well-being-related outcomes has brought attachment theory research into the domain of specific “contexts” (especially achievement domains—like school and sport) in children’s lives. For example, a few researchers have examined the influence of father-child/parent-adolescent attachment relationships on school-related outcomes [76], sport involvement [23], sport friendship [48], psychological need satisfaction and motivation in physical activity [77], and the frequency of physical activity and physical self-concept [78]. However, no research to date has explored variation in attachment characteristics (and associated outcomes) within parent-child relationships, across contexts, and in relation to “episodic” and “global” hierarchical orientations as well. Existing contextual research (e.g., [76,78,79,80]) has mostly used child-parent attachment patterns on a global-level to predict “context-specific” psychological outcomes. It is interesting to speculate whether attachment schemata in relation to a specific attachment figure might be different across contexts and what the potential consequences of this might be. 

Context-specific attachment representations may offer an interesting way of exploring whether and how children are able to separate out, filter, or process parental attachment behavior, differentiating across various context-specific working models. We do not know, at present, whether children do this, whether it is helpful, how it operates, and what the consequences might be. Additionally, according to our earlier conceptualization of a multilevel model (see Figure 1), a person’s contextual attachment working models would presumably share variance with global, episodic, and even other context-specific models and the nature of this variation remains to be unraveled. Contextual attachment representations may be promising ways to expand our understanding of parent-child relationships in specific contexts and in general. 

Theoretically, context-specific variation in attachment patterns offers interesting possibilities for exploring other aspects of attachment. Girme et al.’s [81] recent study indicated that individuals with greater fluctuation (variation in attachment security) within relationship-specific figures showed decreased levels of relationship satisfaction and increased levels of relationship distress over time, especially for “securely” attached individuals who “expected” greater stability within a specific relationship. It seems that future studies could transfer this idea to within-relationship fluctuation by context, exploring whether fluctuation of child-parent attachment security across contexts has a similar detrimental effect on children’s well-being. For example, compared to secure or “organized-insecure” attachment (i.e., anxious/ambivalent, avoidant) models, children with “disorganized/disoriented” attachment patterns have trouble gauging whether proximity seeking and emotional support is a viable or unviable option on any level [82]. Such children are likely to suffer from a breakdown of organized attachment strategies (e.g., primary, hyper-activation, deactivation) because of disorganized, unusual fluctuation between anxiety and avoidance (e.g., [83,84,85]). It may be that some children experience greater variation in attachment security and caregiving behavior from parents across contexts and are consequently more likely to develop globally disorganized attachment representations. Understanding how this variation in context-specific attachment representations within specific parental relationships contributes to inhibiting organized attachment models (and disrupts well-being due to contextual variation) would be an interesting development. In this sense, it would facilitate new ways of examining how context-specific levels of attachment might impact higher-order global levels. That is, perhaps context-specific variation within a parent makes it harder for individuals to crystalize established generalizations about the given attachment figure. This would suggest that contextual fluctuation is an inhibitory factor in higher-order generalizations of attachment. Investigation of such new hypotheses would be permitted by exploring the idea of contextual attachment variation.

## 5. Conclusions

What is the nature of child-parent attachment models across different contexts? What might the relationships between episodic, contextual (e.g., sport-specific, school-specific), and global attachment representations with parents look like in a hierarchical sense? What other possible contexts (apart from “sport” and “school”) might exist within parent-child relationships and how do we identify what a context “is”? Could the conceptualization of contextual child-parent attachment generalize to other relationship-specific partners (e.g., close friends, romantic partners, teachers, coaches), and if it could, what would contextual attachment mean within these specific relationships? These major conceptual questions are considerably complex and further research is needed to validate and explore “context-specific” attachment characteristics on many levels.

This article attempts to shed light on a potentially unexplored area of attachment theory by forwarding the idea of contextual attachment within parent-child relationships. It should be acknowledged that our initial discussions of plausible attachment contexts have been based on a view of Western children’s lives and family structure. It should be noted that cultural differences between children from all sorts of backgrounds (e.g., Western working and middle-class, non-Western, rural eco-social environments) also merit significant discussion in relation to the concept of contextual attachment. Understanding the various cultural and sub-cultural differences that exist in relation to within-person attachment contexts will be an important avenue of future research. Perhaps, for example, in other cultures it is not expected that a single attachment figure would be “involved” significantly in the different contexts that make up children’s lives. Perhaps omnipotent involvement in multiple child life contexts is more relevant to certain cultures than others, making context-specific attachment more relevant to these cultures than others. Furthermore, perhaps within-person contextual attachment variation applies to multiple attachment figures as well. It may be that all attachment relationships are context-specific and that children engage in fluid relational interactions between different attachment figures and within-different attachment figures from context to context. This would make the organization of attachment-related life a highly complex and fluid dynamic to understand.

It is also important to note that research into attachment representations across different relationship referents (e.g., parents, peers, romantic partners) has suggested that it is important to recognize the significance of the hierarchical importance of such attachment representations (e.g., [43]). In this sense, it is likely that in an abstract sense people “weigh” the relative importance of the relationships in their lives and this may determine the impact specific attachment representations have on personality and well-being. Similarly, perhaps different contextual attachment representations within specific relationships also have relative importance and can be hierarchically ordered. That is, perhaps (for a variety of reasons) a child might place more hierarchical importance on the sporting interactions they have with their parents, rendering this contextual attachment representation more significant and important in shaping their overall representation of their parent. Such ideas about relative importance of various contextual attachment representations merit further exploration. It may also be the case that different contexts expose children to different attachment representations with multiple figures (e.g., a child may experience her mother as insecure in sport and her father as secure in sport). In this case, the relative importance of different attachment figures in a given context may hold sway over which model influences context-specific psychological outcomes.

Future researchers will need to pay particular attention to the issue of how to empirically research and “measure” one’s “context-specific” attachment orientations with a given relationship. We noted at the beginning of this paper that the attachment research has diverged into two major schools of thought: the psychodynamic, clinical school, and the personality and social psychology school. Much of the research that we have connected our ideas to in this paper stems from assumptions made by the personality and social psychology tradition. We have advocated and relied upon assumptions that lend themselves easily to a self-report paradigm, and it would seem logical and expedient to investigate context-specific attachment through the development of self-report items designed to tap into within-person variation between contexts. Such self-report measures may begin to permit measurement of how specific attachment figures are experienced (in relation to security and insecurity) within specific contexts. These perceptions can then be explored in relation to how they relate to each other (e.g., are context-specific attachment perceptions radically different within a given parent? Or are they similar?), whether this matters (e.g., does it matter whether children experience their parents differently across contexts? And why?), and how such context-specific perceptions fit into the broader hierarchical organization of attachment models. However, it is also important to think beyond this and to explore the possibility of exploring within-person attachment variation using assessment tools that move beyond self-report and focus upon issues such as (a) a deeper qualitative exploration of the meaning and experience of within-person contextual variation, (b) how subconscious processing and characteristics are orchestrated contextually, and (c) whether attachment figures themselves are aware of the contextual fluctuation detected by children. It is important to evolve this area of research in a broader sense than self-report alone would permit. 

## References

[1] Bowby J. (1970). Attachment and Loss: Volume I. Attachment.

[2] Mikulincer M., Shaver P.R., Mikulincer M., Shaver P.R. (2014). Mechanisms of Social Connection: From Brain to Group.

[3] Sroufe L.A.E. (1977). Attachment as an Organizational Construct. Child Dev..

[4] Mikulincer M., Shaver P.R., Sapir-Lavid Y., Avihou-Kanza N. (2009). What’s inside the minds of securely and insecurely attached people? The secure-base script and its associations with attachment-style dimensions. J. Personal. Soc. Psychol..

[5] Cassidy J., Kobak R.R. (2001). Avoidance and its relationship with other defensive processes. Clinical Implications of Attachment.

[6] Main M. (1990). Cross-Cultural Studies of Attachment Organization: Recent Studies, Changing Methodologies, and the Concept of Conditional Strategies. Hum. Dev..

[7] Ainsworth M.D.S. (1967). Infancy in Uganda: Infant Care and the Growth of Love.

[8] Ainsworth M.D.S. (1978). Patterns of Attachment: A Psychological Study of the Strange Situation.

[9] Carr S., Colthurst K., Coyle M., Elliott D. (2013). Attachment dimensions as predictors of mental health and psychosocial well-being in the transition to university. Eur. J. Psychol. Educ..

[10] Jacobvitz D., Curran M., Moller N. (2002). Measurement of adult attachment: The place of self-report and interview methodologies. Attach. Hum. Dev..

[11] Bartholomew K., Shaver P.R., Simpson J.A., Rholes W.S. (1998). Methods of assessing adult attachment: Do they converge?. Ttachment Theory and Close Relationships.

[12] Carr S. (2012). Attachment in Sport, Exercise and Wellness.

[13] Fraley R.C. (2002). Attachment Stability from Infancy to Adulthood: Meta-Analysis and Dynamic Modeling of Developmental Mechanisms. Personal. Soc. Psychol. Rev..

[14] Klohnen E.C., Bera S. (1998). Behavioral and experiential patterns of avoidantly and securely attached women across adulthood: A 31-year longitudinal perspective. J. Personal. Soc. Psychol..

[15] Simpson J.A., Collins W.A., Tran S., Haydon K.C. (2007). Attachment and the experience and expression of emotions in romantic relationships: A developmental perspective. J. Personal. Soc. Psychol..

[16] Dykas M.J., Cassidy J. (2011). Attachment and the processing of social information across the life span: Theory and evidence. Psychol. Bull..

[17] Bowlby J. (1973). Attachment and Loss: Volume II. Separation: Anxiety and Anger.

[18] Crowell J., Waters E. (2005). Attachment Representations, Secure-Base Behavior, and the Evolution of Adult Relationships: The Stony Brook Adult Relationship Project. Attachment from Infancy to Adulthood: The Major Longitudinal Studies.

[19] Mikulincer M., Shaver P.R. (2016). Attachment in Adulthood: Structure, Dynamics, and Change.

[20] Owens G., Crowell J.A., Pan H., Treboux D., O’Connor E., Waters E. (1995). The Prototype Hypothesis and the Origins of Attachment Working Models: Adult Relationships with Parents and Romantic Partners. Monogr. Soc. Res. Child Dev..

[21] Fraley R.C., Shaver P.R., Cassidy J., Shaver P.R. (1999). Loss and bereavement: Attachment theory and recent controversies concerning “grief work” and the nature of detachment. Handbook of Attachment: Theory, Research, and Clinical Applications.

[22] Shaver P.R., Belsky J., Brennan K.A. (2000). The adult attachment interview and self-reports of romantic attachment: Associations across domains and methods. Pers. Relatsh..

[23] Sukys S., Lisinskiene A., Tilindiene I. (2015). Adolescents’ Participation in Sport Activities and Attachment to Parents and Peers. Soc. Behav. Personal. Int. J..

[24] Mercer J. (2011). Attachment theory and its vicissitudes: Toward an updated theory. Theory Psychol..

[25] Seibert A.C., Kerns K.A. (2009). Attachment figures in middle childhood. Int. J. Behav. Dev..

[26] Furman W., Buhrmester D. (2009). The Network of Relationships Inventory: Behavioral Systems Version. Int. J. Behav. Dev..

[27] Hazan C., Zeifman D., Bartholomew K., Perlman D. (1994). Sex and the psychological tether. Advances in Personal Relationships, Vol. 5. Attachment Processes in Adulthood.

[28] Fraley R.C., Davis K.E. (1997). Attachment formation and transfer in young adults’close friendships and romantic relationships. Pers. Relatsh..

[29] Mayselessx O. (2004). Home Leaving to Military Service. J. Adolesc. Res..

[30] Zhang H., Chan D.K.-S., Teng F. (2011). Transfer of Attachment Functions and Adjustment Among Young Adults in China. J. Soc. Psychol..

[31] Hazan C., Zeifman D., Cassidy J., Shaver P.R. (1999). Pair bonds as attachments: Evaluating the evidence. Handbook of Attachment: Theory, Research, and Clinical Applications.

[32] Sage G., Sage G. (1980). Study of social aspects of sport. Sport in American Society: Selected Readings.

[33] van Ijzendoorn M.H., Sagi-Schwartz A., Cassidy J., Shaver P.R. (2008). Cross-cultural patterns of attachment: Universal and contextual dimensions. Handbook of Attachment: Theory, Research, and Clinical Applications.

[34] Allen J.P., Shaver J., Cassidy P.R. (2008). The attachment system in adolescence. Handbook of Attachment: Theory, Research, and Clinical Applications.

[35] Trinke S.J., Bartholomew K. (1997). Hierarchies of Attachment Relationships in Young Adulthood. J. Soc. Pers. Relat..

[36] Schachner D.A., Shaver P.R., Gillath O. (2008). Attachment style and long-term singlehood. Pers. Relatsh..

[37] Overall N.C., Fletcher G.J.O., Friesen M.D. (2003). Mapping the Intimate Relationship Mind: Comparisons between Three Models of Attachment Representations. Personal. Soc. Psychol. Bull..

[38] Lewis M. (1994). Does Attachment Imply a Relationship or Multiple Relationships?. Psychol. Inq..

[39] Pierce T., Lydon J.E. (2001). Global and specific relational models in the experience of social interactions. J. Personal. Soc. Psychol..

[40] La Guardia J.G., Ryan R.M., Couchman C.E., Deci E.L. (2000). Within-person variation in security of attachment: A self-determination theory perspective on attachment, need fulfillment, and well-being. J. Personal. Soc. Psychol..

[41] Collins N.L., Read S.J., Bartholomew K., Perlman D. (1994). Cognitive representations of attachment: The structure and function of working models. Advances in Personal Relationships, Vol. 5. Attachment Processes in Adulthood.

[42] Imamoğlu S., Imamoğlu E.O. (2006). Relationship Between General and Context-Specific Attachment Orientations in a Turkish Sample. J. Soc. Psychol..

[43] Gillath O., Karantzas G.C., Fraley R.C. (2016). Adult Attachment: A Concise Introduction to Theory and Research a Concise Introduction to Theory and Research.

[44] Collins N.L., Allard L.M., Fletcher G.J.O., Clark M.S. (2001). No TitleCognitive representations of attachment: The content and function of working models. Blackwell Handbook of Social Psychology: Vol. 2. Interpersonal Processes.

[45] Vallerand R.J. (1997). Toward a hierarchical model of intrinsic and extrinsic motivation. Adv. Exp. Soc. Psychol..

[46] Vallerand R.J., Hagger M.S. (2007). A hierarchical model of intrinsic and extrinsic motivation for sport and physical activity. Intrinsic Motivation and Self-Determination in Exercise and Sport.

[47] Furman W., Belle D. (1989). The development of children’s social networks. Wiley Series on Personality Processes. Children’s Social Networks and Social Supports.

[48] Carr S. (2009). Adolescent-parent attachment characteristics and quality of youth sport friendship. Psychol. Sport Exerc..

[49] Weigand D., Carr S., Petherick C., Taylor A. (2001). Motivational climate in sport and physical education: The role of significant others. Eur. J. Sport Sci..

[50] Eccles J.S., Adler T.F., Futterman R., Goff S.B., Kaczala C.M., Meece J.L., Midgley C., Spence J.T. (1983). Expectancies, values, and academic behaviors. Expectancies, Values, and Academic Behaviors.

[51] Cox A.E., Whaley D.E. (2004). The Influence of Task Value, Expectancies for Success, and Identity on Athletes’ Achievement Behaviors. J. Appl. Sport Psychol..

[52] Dietrich J., Viljaranta J., Moeller J., Kracke B. (2017). Situational expectancies and task values: Associations with students’ effort. Learn. Instr..

[53] Jambor E.A. (1999). Parents Children’s Socializing Agents in Youth Soccer. J. Sport Behav..

[54] Greendorfer S.L., Lewko J.H. (1978). Role of Family Members in Sport Socialization of Children. Res. Q. Am. Alliance Health Phys. Educ. Recreat..

[55] Carr S., Weigand D., Papaioannou A.G., Hackfort D. (2014). Families. Routledge Companion to Sport and Exercise Psychology: Global Perspectives and Fundamental Concepts.

[56] Fredricks J.A., Eccles J.S., Weiss M.R. (2004). Parental Influences on Youth Involvement in Sports. Developmental Sport and Exercise Psychology: A Lifespan Perspective.

[57] Eccles J.S., Wigfield A., Schiefele U., Damon W., Eisenberg N. (1998). Motivation to succeed. Handbook of Child Psychology: Social, Emotional, and Personality Development.

[58] Tofler I.R., Knapp P.K., Lardon M.T. (2005). Achievement by Proxy Distortion in Sports: A Distorted Mentoring of High-Achieving Youth. Historical Perspectives and Clinical Intervention with Children, Adolescents, and their Families. Clin. Sports Med..

[59] Assor A., Roth G., Deci E.L. (2004). The Emotional Costs of Parents’ Conditional Regard: A Self-Determination Theory Analysis. J. Personal..

[60] Ames C. (1992). Classrooms: Goals, structures, and student motivation. J. Educ. Psychol..

[61] Brophy J. (1987). Synthesis of Research on Strategies for Motivating Students to Learn. Educ. Leadersh..

[62] Weiss M.R., Amorose A.J., Wilko A.M. (2009). Coaching Behaviors, Motivational Climate, and Psychosocial Outcomes among Female Adolescent Athletes. Pediatr. Exerc. Sci..

[63] Hall H.K., Kerr A.W. (1997). Motivational Antecedents of Precompetitive Anxiety in Youth Sport. Sport Psychol..

[64] White S.A., Zellner S.R. (1996). The Relationship between Goal Orientation, Beliefs about the Causes of Sport Success, and Trait Anxiety among High School, Intercollegiate, and Recreational Sport Participants. Sport Psychol..

[65] Rapport L.J., Meleen M. (1998). Childhood Celebrity, Parental Attachment, and Adult Adjustment: The Young Performers Study. J. Personal. Assess..

[66] Weiss M.R., Fretwell S.D. (2005). The Parent-Coach/Child-Athlete Relationship in Youth Sport. Res. Q. Exerc. Sport.

[67] Tofler I.R., Butterbaugh G.J. (2005). Developmental overview of child and youth sports for the twenty-first century. Clin. Sports Med..

[68] Baldwin M.W. (1994). Primed Relational Schemas as a Source of Self-Evaluative Reactions. J. Soc. Clin. Psychol..

[69] Deci E.L., Ryan R.M. (1995). Human Autonomy. Efficacy, Agency, and Self-Esteem.

[70] Harter S. (1993). Causes and Consequences of Low Self-Esteem in Children and Adolescents. Self-Esteem.

[71] Assor A., Tal K. (2012). When parents’ affection depends on child’s achievement: Parental conditional positive regard, self-aggrandizement, shame and coping in adolescents. J. Adolesc..

[72] Assor A., Kanat-Maymon Y., Roth G., Weinstein N. (2014). Parental conditional regard: Psychological costs and antecedents. Human Motivation and Interpersonal Relationships.

[73] Assor A., Vansteenkiste M., Kaplan A. (2009). Identified versus introjected approach and introjected avoidance motivations in school and in sports: The limited benefits of self-worth strivings. J. Educ. Psychol..

[74] Assor A., Mikulincer M., Shaver P.R. (2011). Autonomous moral motivation: Consequences, socializing antecedents, and the unique role of integrated moral principles. The Social Psychology of Morality.

[75] Ryan R.M., Deci E.L., Grolnick W.S., Cicchetti D., Cohen D.J. (1995). Autonomy, relatedness, and the self: Their relation to development and psychopathology. Wiley Series on Personality Processes. Developmental Psychopathology, Vol. 1. Theory and Methods.

[76] Newland L.A., Chen H.-H., Coyl-Shepherd D.D. (2013). Associations Among Father Beliefs, Perceptions, Life Context, Involvement, Child Attachment and School Outcomes in the U. S. and Taiwan. Fathering.

[77] Ullrich-French S., Smith A.L., Cox A.E. (2011). Attachment relationships and physical activity motivation of college students. Psychol. Health.

[78] Li R., Bunke S., Psouni E. (2016). Attachment relationships and physical activity in adolescents: The mediation role of physical self-concept. Psychol. Sport Exerc..

[79] Felton L., Jowett S. (2013). Attachment and well-being: The mediating effects of psychological needs satisfaction within the coach–athlete and parent–athlete relational contexts. Psychol. Sport Exerc..

[80] Felton L., Jowett S. (2013). The mediating role of social environmental factors in the associations between attachment styles and basic needs satisfaction. J. Sports Sci..

[81] Girme Y.U., Agnew C.R., VanderDrift L.E., Harvey S.M., Rholes W.S., Simpson J.A. (2018). The ebbs and flows of attachment: Within-person variation in attachment undermine secure individuals’ relationship wellbeing across time. J. Personal. Soc. Psychol..

[82] Main M., Solomon J., Greenberg M.T., Cicchetti D.E.M.C. (1990). Procedures for identifying infants as disorganized/disoriented during the Ainsworth Strange Situation. The John D. and Catherine T. MacArthur Foundation Series on Mental Health and Development. Attachment in the Preschool Years: Theory, Research, and Intervention.

[83] Hesse E., Cassidy J., Shaver P.R. (2008). The Adult Attachment Interview: Protocol, method of analysis, and empirical studies. Handbook of Attachment: Theory, Research, and Clinical Applications.

[84] Hesse E., Main M. (2000). Disorganized Infant, Child, and Adult Attachment: Collapse in Behavioral and Attentional Strategies. J. Am. Psychoanal. Assoc..

[85] Lyons-Ruth K., Jacobvitz D., Cassidy J., Shaver P.R. (2008). Attachment disorganization: Genetic factors, parenting contexts, and developmental transformation from infancy to adulthood. Handbook of Attachment: Theory, Research, and Clinical Applications.

